# Brachytherapy With Modified Rotte-y Applicator for Inoperable Endometrial Cancer: A Case Report

**DOI:** 10.7759/cureus.72462

**Published:** 2024-10-27

**Authors:** Rashi Agrawal, Nelesh Aggarwal, Swasti Swasti, Kailash Mittal, Arnav Sharma

**Affiliations:** 1 Radiation Oncology, Max Super Speciality Hospital, Ghaziabad, IND; 2 Gynecologic Oncology, Max Super Speciality Hospital, Ghaziabad, IND; 3 Radiology and Radiation Oncology, Uttar Pradesh University of Medical Sciences, Etawah, IND

**Keywords:** brachytherapy, carcinoma endometrium, gynae-oncology, intracavitary, rotte-y applicator

## Abstract

The preferred treatment for medically inoperable early-stage endometrial cancer (EC) is definitive radiation therapy (RT), either in the form of brachytherapy (BT) alone or in combination with external beam radiotherapy (EBRT). This case report details a modified brachytherapy approach for a 59-year-old female patient who was known to have multiple comorbidities, poor performance status, and progressive slowness in activities of daily living. She presented with a complaint of post-menopausal bleeding per vaginum, for which she underwent investigations and was diagnosed with a case of endometrial adenocarcinoma, International Federation of Gynecology and Obstetrics (FIGO) stage IA and grade I. After the tumor board discussion, the patient was deemed medically inoperable and planned for radical radiotherapy. She received EBRT via the image-guided intensity-modulated radiotherapy technique, a dose of 50.4 Gy in 28 fractions to the uterus, cervix, and upper 2 cm of the vagina, followed by weekly intracavitary brachytherapy (ICBT), a dose of 4.5 Gy in the first fraction via the Rotte-Y applicator and 5.2 Gy in the second and third fractions each via the modified Rotte-Y applicator. The modified applicator demonstrated better coverage of the target with a lesser dose to the organs at risk (OARs) and underscores its potential use in the future for such cases.

## Introduction

Endometrial cancer (EC) is a major health concern, ranking as the sixth most common cancer among females globally [1]. This malignancy significantly impacts women’s health, particularly in developed nations where its prevalence is notably high. In contrast, while the incidence rate of EC in India remains relatively low compared to Western countries, it is experiencing a gradual increase. This upward trend can be attributed to the growing prevalence of obesity and an aging population, both of which are risk factors for EC. For patients with EC, the initial standard treatment involves surgical intervention. This is followed by adjuvant radiation therapy (RT), which may be administered in the form of brachytherapy (BT) or external beam radiation therapy (EBRT), depending on individual clinical indications. However, for patients who are not candidates for surgery, definitive RT is the preferred treatment modality [2].

In the context of definitive RT, the typical protocol involves a combination of EBRT and BT, with the Rotte-Y applicator being the conventional choice for BT. Despite its widespread use, the Rotte-Y applicator has limitations, particularly in patients with a large uterine body. It may not provide sufficient dosimetric coverage, which can compromise treatment efficacy. To address this, several three-channel BT applicators have been developed to enhance dosimetric coverage for patients with larger uterine volumes [3,4]. These applicators aim to improve treatment outcomes by delivering more precise radiation doses to the target area while minimizing exposure to surrounding healthy tissues. However, the availability of these specialized three-channel BT applicators is limited, especially in regions where the proportion of inoperable EC cases is relatively low. This commercial scarcity can pose challenges in managing patients who require these advanced applicators for optimal treatment.

We present a case involving a medically inoperable Indian patient treated using an innovative approach. This technique combines the standard Rotte-Y applicator with the Fletcher 30° standard tandem (Elekta Corp., Stockholm, SWE), both of which are commercially available and commonly used. This modified approach not only aims to improve dosimetric coverage but also seeks to mitigate the gastrointestinal toxicities that are associated with the standard Rotte-Y applicator.

## Case presentation

**Figure 1:**
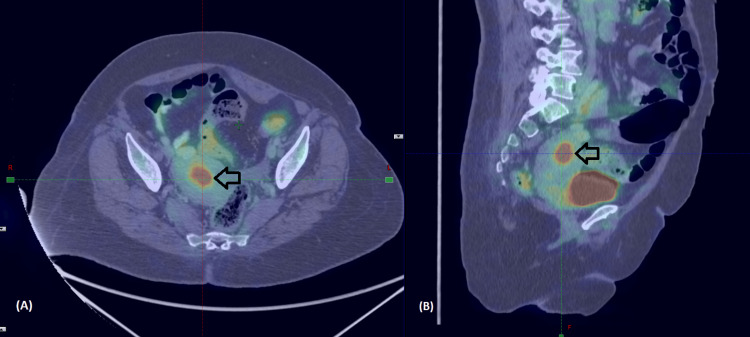
Axial (A) and sagittal (B) sections of PET-CECT showing intrauterine lesion PET-CECT: Positron emission tomography and contrast-enhanced computed tomography

A 59-year-old postmenopausal female with hypertension, diabetes, parkinsonism, and osteoarthritis presented with complaints of postmenopausal bleeding. She had a poor performance status (Karnofsky performance score (KPS) = 40), with progressive slowness in activities of daily living and difficulty swallowing and speaking. She was evaluated with an endometrial biopsy, which revealed predominant atypical endometrial hyperplasia with foci of endometrioid endometrial adenocarcinoma, grade I, with no lympho-vascular invasion. A positron emission tomography and contrast-enhanced computed tomography (PET-CECT) scan showed a fluorodeoxyglucose (FDG)-avid (maximum standardized uptake value (SUV max): 5.3), heterogeneously enhancing 2.6 cm x 2.2 cm intrauterine lesion in the fundus and body of the uterus, with less than half of myometrial involvement (Figure 1). Due to logistical issues (financial constraints), an MRI could not be performed. Clinically, this was an International Federation of Gynecology and Obstetrics (FIGO) 2009-stage IA disease.

**Figure 2:**
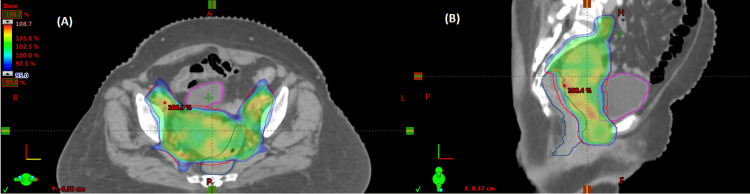
Axial (A) and sagittal (B) sections of the EBRT plan show 95% dose color-wash covering the target EBRT: External beam radiotherapy

The case was discussed by the tumor board, and given the multiple comorbidities and low-performance status, the patient was deemed medically inoperable. Since comprehensive staging with MRI was unavailable, the patient was planned for definitive EBRT followed by intracavitary brachytherapy (ICBT) [5]. Based on the linear quadratic model, the total planned biologically equivalent dose in 2 Gy fractions (EQD2) for EBRT plus ICBT was 70 Gy, assuming an α/β ratio of 10. A dose of 50.4 Gy was delivered to the uterus, cervix, upper 2 cm of the vagina, and regional lymph nodes in 28 fractions in 37 days (five fractions per week) using the image-guided intensity-modulated RT technique (Figure 2). The ICBT dose of 5.2 Gy per fraction for three fractions (delivered weekly), prescribed 2 cm from the central axis at the midpoint along the uterine applicator, was planned to cover the entire uterus and cervix (vagina was not included in the BT boost due to disease location and potential toxicity), using the standard Rotte-Y applicator [6].

The applicator was placed under spinal anesthesia in strict aseptic conditions and secured in position with vaginal packing using ribbon gauze and a T-bandage. An RT-planning CT scan was performed. The ICBT plan was made according to the prescription point, and graphical optimization was done to reduce the dose to organs at risk (OARs) without compromising uterine coverage. However, the OARs doses remained very high. As a result, the planned dose was decreased to 4.5 Gy for the first fraction.

**Figure 3:**
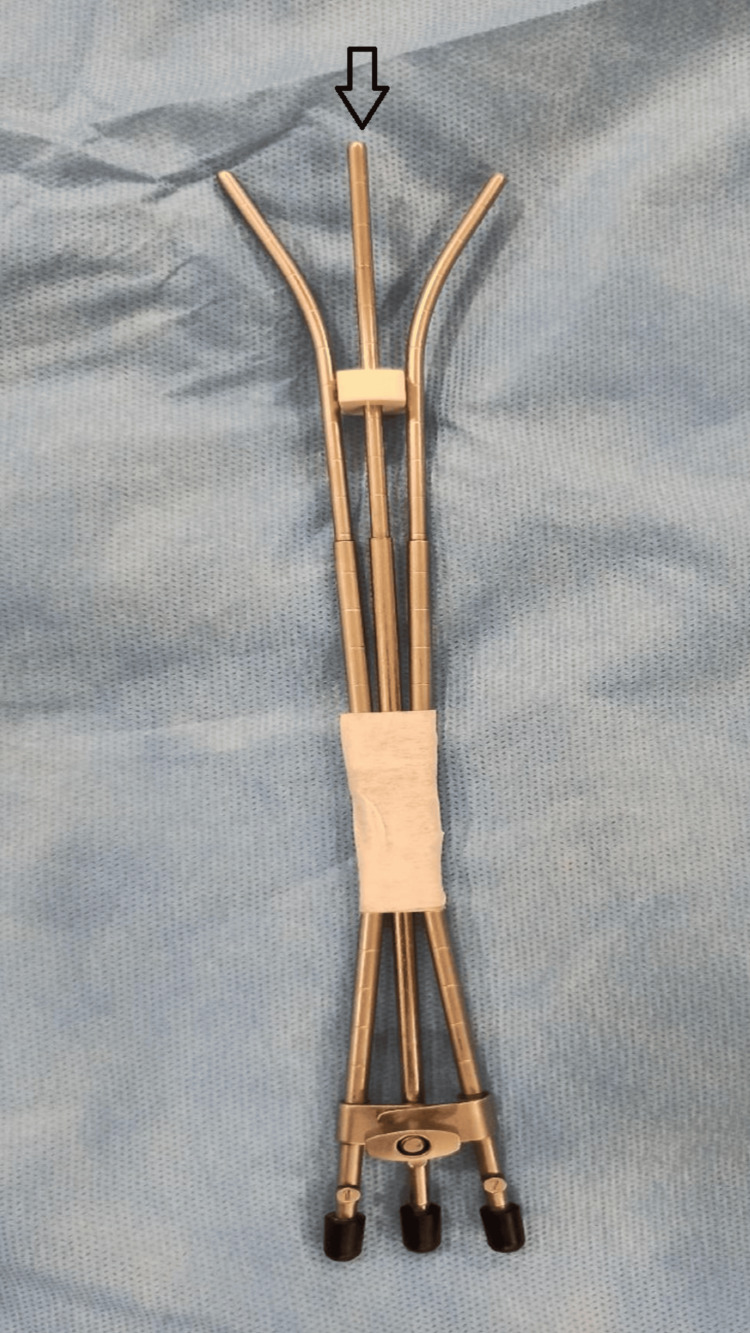
Photograph of the Rotte-Y applicator combined with Fletcher 30° standard central tandem and flange (arrow)

The next two fractions (second and third) were administered using a modified Rotte-Y applicator approach, which combined the Rotte-Y applicator with the Fletcher 30° standard central tandem. A misoprostol suppository (200 mcg) was used per vaginum for cervical dilation 1.5 hours before applicator placement [7]. The length of the central tandem was fixed at the 6 cm mark using the flange to prevent uterine perforation during applicator maneuvering. The central tandem was positioned under the Rotte-Y applicator to fit into its groove, and further immobilization of the tandems was achieved by taping them together (Figure 3).

**Figure 4:**
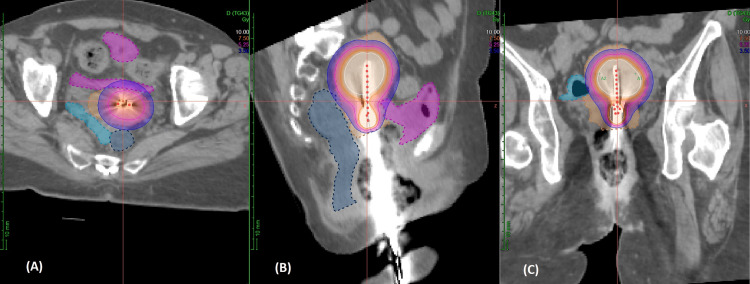
Axial (A), sagittal (B), and coronal (C) sections of CT-based planning for BT using the modified Rotte-Y applicator The prescription isodose (5.2Gy) line is pink, and the 3.5Gy isodose line is blue. The uterus and cervix are contoured in orange color. BT: Brachytherapy

A CT-based BT planning was done on the Oncentra Brachy treatment planning system/module (Elekta Corp., Stockholm, SWE) in each session, and remote afterloading was done using Ir-192 with the microSelectron afterloader (Elekta Corp., Stockholm, SWE). Figure 4 shows the iso-dose line of 5.2 Gy covering the uterus and cervix when the modified applicator was used. The uterus and cervix were contoured to assess the dosimetric coverage provided by the 2 cm prescription point. The dosimetric coverage of the uterus slightly improved along with a reduction in dose to the OARs in the modified sessions, as shown in Table 1. Based on the linear quadratic model, the total EQD2 received for EBRT plus BT was 68.2 Gy, assuming an α/β ratio of 10.

**Table 1:** The dosimetry parameters of BT sessions BT: Brachytherapy, CTV: Clinical target volume

	1^st^ fraction of BT	2^nd^ fraction of BT	3^rd^ fraction of BT
Prescribed dose	4.5 Gy	5.2 Gy	5.2 Gy
Bladder D2cc (%)	5.2 Gy (115.0%)	4.5 Gy (86.73%)	3.5 Gy (66.53%)
Rectum D2cc (%)	2.8 Gy (61.77%)	1.9 Gy (36.92%)	2.7 Gy (51.73%)
Sigmoid D2cc (%)	3.6 Gy (79.11%)	4.5 Gy (86.73%)	3.6 Gy (69.23%)
CTV volume	194cc	186cc	198cc
CTV D90%	2.71 Gy	2.69 Gy	3.46Gy

**Figure 5:**
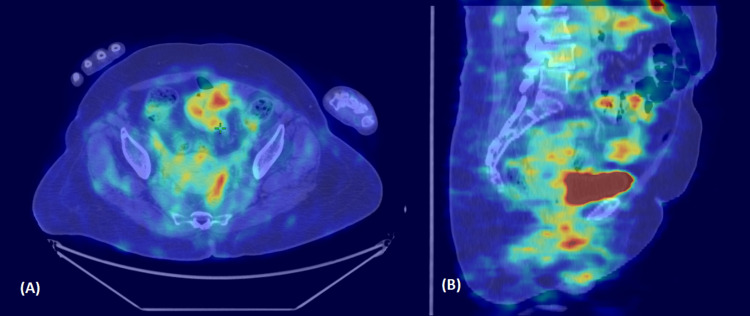
Axial (A) and sagittal (B) images of PET-CECT one year after completing RT PET-CECT: Positron emission tomography and contrast-enhanced computed tomography, RT: Radiation therapy

The follow-up PET-CECT performed four months after RT showed that the tumor had regressed and was considered a complete response. An MRI of the pelvis could not be done at that time due to logistical issues. The second follow-up, which included both a PET-CT and an MRI of the pelvis one year after completing RT, revealed no abnormal findings, and the response was again considered complete (Figure 5). The patient was asymptomatic and did not report any grade 3 or 4 acute or late toxicity. Due to poor performance status, grade 1 or 2 toxicities were not evaluable.

## Discussion

Surgery is typically the first line of treatment for patients with early-stage EC. However, due to an aging population and an increased prevalence of comorbidities, the number of inoperable cases is rising. For these medically inoperable patients, definitive RT is the preferred treatment. A recent retrospective analysis of 103 inoperable EC patients concluded that EBRT combined with BT provides good two- and five-year cancer-specific survival rates of 88.7% and 71.2%, respectively, with the best outcomes observed in stages I-II [8].

The use of modified Heyman applications [9], first described in the early 1970s, is a time-consuming procedure that is not commonly practiced. Consequently, the Rotte-Y applicator is frequently used for delivering BT to patients with EC. A study evaluating the 10-year treatment outcomes of using the Rotte-Y applicator in 49 inoperable EC patients reported three- and five-year actuarial cause-specific survival rates of 93% and 87%, respectively, with only 13% of patients experiencing grade ≥ 2 late toxicity. The study concluded that incorporating three-dimensional treatment planning could further reduce treatment-related morbidities [10]. However, with 3D planning and image-guided BT, the dosimetric coverage provided by the standard Rotte-Y applicator was found to be insufficient, leading to the development of three-channel intrauterine devices.

Various three-channel intrauterine applicators have been used for BT in patients with inoperable EC, achieving excellent dose distribution for the uterine body [3,4]. Johnson et al. employed a commercially available three-channel endometrial applicator to treat intact uterine cancer. Eighteen treatment plans using single, dual, and triple tandems were created for three patients. The three-tandem applicator provided superior uterine coverage compared to single or dual-tandem applicators in the chosen patient [11].

In this case, the Rotte-Y applicator and Fletcher regular tandem were combined to create a three-channel applicator quickly. Adequate cervical dilatation is required for this modified applicator and was achieved by using misoprostol, as recommended by Ibrahim et al. [7]. Dosimetric improvements for the large uterine body were observed with this modified applicator without increasing the dose to the OARs beyond levels administered with the Rotte-Y applicator alone. In a further literature review, a similar combination approach has been reported by Takagawa et al. [12]. However, they used a whole pelvic three-dimensional conventional RT field with a midline block and prescribed a dose of 6 Gy per fraction (four fractions) in the BT treatment plan as compared to our treatment strategy.

Compared to the traditional Rotte-Y applicator, three-channel BT applicators offer superior dosimetric coverage for the uterus and reduce toxicity to the OARs for patients with a large uterine body. Therefore, for facilities lacking a commercially available three-channel endometrial applicator, this technique provides an alternative option for three-channel BT in EC therapy. This method is not suitable for EC patients with narrow cervical canals, as adequate cervical dilatation is essential. Additionally, the potential for uterine perforation must be considered, particularly when treating older adult patients with weakened uterine walls.

## Conclusions

External beam radiotherapy combined with BT is a good curative option in inoperable EC stages I to III. For BT, benefit can be obtained from this modified approach, which combines the Rotte-Y applicator with the Fletcher regular tandem in patients with big uteri. However, adequate cervical dilatation is required to implant the tandem and Rotte-Y applicator simultaneously. The case demonstrates the potential for adapting existing technologies to meet the needs of specific patient populations but also underscores the importance of continued research and innovation in the field of radiation oncology. By optimizing treatment techniques, we can better address the evolving challenges of managing EC in diverse clinical settings and improve patient outcomes.
